# Machine Learning Assisted Handheld Confocal Raman Micro-Spectroscopy for Identification of Clinically Relevant Atopic Eczema Biomarkers

**DOI:** 10.3390/s22134674

**Published:** 2022-06-21

**Authors:** Kapil Dev, Chris Jun Hui Ho, Renzhe Bi, Yik Weng Yew, Dinish U. S, Amalina Binte Ebrahim Attia, Mohesh Moothanchery, Steven Thng Tien Guan, Malini Olivo

**Affiliations:** 1 Translational Biophotonics Lab, Institute of Bioengineering and Bioimaging, Agency for Science, Technology and Research (A*STAR), Singapore 138667, Singapore; kapil_dev@ibb.a-star.edu.sg (K.D.); chris.hojunhui@gmail.com (C.J.H.H.); bi_renzhe@ibb.a-star.edu.sg (R.B.); dinish@ibb.a-star.edu.sg (D.U.S.); amalina_attia@ibb.a-star.edu.sg (A.B.E.A.); moheshm@gmail.com (M.M.); 2 National Skin Centre, Singapore 308205, Singapore; ywyew@nsc.com.sg (Y.W.Y.); steventhng@nsc.com.sg (S.T.T.G.)

**Keywords:** atopic eczema, confocal Raman spectroscopy, partial least squares discriminant analysis, variable importance in projection

## Abstract

Atopic dermatitis (AD) is a common chronic inflammatory skin dermatosis condition due to skin barrier dysfunction that causes itchy, red, swollen, and cracked skin. Currently, AD severity clinical scores are subjected to intra- and inter-observer differences. There is a need for an objective scoring method that is sensitive to skin barrier differences. The aim of this study was to evaluate the relevant skin chemical biomarkers in AD patients. We used confocal Raman micro-spectroscopy and advanced machine learning methods as means to classify eczema patients and healthy controls with sufficient sensitivity and specificity. Raman spectra at different skin depths were acquired from subjects’ lower volar forearm location using an in-house developed handheld confocal Raman micro-spectroscopy system. The Raman spectra corresponding to the skin surface from all the subjects were further analyzed through partial least squares discriminant analysis, a binary classification model allowing the classification between eczema and healthy subjects with a sensitivity and specificity of 0.94 and 0.85, respectively, using stratified K-fold (K = 10) cross-validation. The variable importance in the projection score from the partial least squares discriminant analysis classification model further elucidated the role of important stratum corneum proteins and lipids in distinguishing two subject groups.

## 1. Introduction

Atopic dermatitis (AD) is a chronic disease with a current prevalence of 10–15% in developed countries. The chronic skin inflammatory disorder affects the quality of life ranging from intense itch to mental health issues such as social impact, anxiety, depression, etc. along with economic healthcare burden in terms of direct and indirect costs [1]. In a recent published survey, it was confirmed that the overall prevalence of AD is 13.1% in a Singaporean population with an even higher prevalence of 20.6% in children [2,3]. It is characterized by human skin epidermal barrier dysfunction culminating in dry skin and immunoglobulin E (IgE)-mediated sensitization to food, external environmental allergens, irritants, etc. [4,5]. AD is primarily a disease of an immune system with cytokines IL-4, IL13, and IL-33 causing skin barrier dysfunction such as increased PH, reduced water retention, regular itch, etc., and hence eczema. The skin of AD patients has been demonstrated to have increased trans-epidermal water loss (TEWL) and defects in the terminal keratinocyte differentiation. The formation and function of the stratum corneum (SC) is controlled by a set of genes, including filaggrin (FLG) which encodes profilaggrin, the precursor of the filament aggregating protein filaggrin [6]. FLG is broken down into amino acids, which are a crucial component in forming the natural moisturizing factor (NMF) and further hydration of the SC [7]. The presence of FLG mutations has been associated with decreased SC hydration and decreased NMF components, as well as increased AD severity [5,6]. Current assessment of eczema severity is subjective and dependent on the clinicians’ judgements on localized skin inflammation, TEWL, and other biophysical measurements and does not consider the concentration of skin constituents. Hence, there is a need for an objective eczema severity scoring method that is easy to measure and yet sensitive to change with minimal bias. Confocal Raman micro-spectroscopy (CRM) allows for non-invasive, in vivo measurements of skin biomolecular composition or constituents up to several hundred micrometers below the skin surface to detect important nucleic acids, proteins, lipids, etc. Raman scattering or Raman spectroscopy is a measure of the inelastic scattering of photons by matter, i.e., there is either an increase or decrease in the scattered photon energy. The SC of the human skin consists of corneocytes surrounded by multilamellar lipid membranes that prevent excessive water loss from the body and entrance of chemical and biochemical substances from the outside environment. Major skin barrier lipids are ceramides, fatty acids, and cholesterol, and its dysfunction in terms of quantitative analysis of the molecular composition of skin has been widely studied using CRM [8,9,10].

Caspers et al. has studied the in vivo and in vitro skin characterization of its molecular composition varying with respect to the depth using CRM. Water, NMF, and other amino acids in the stratum corneum were quantified with the help of Raman spectra from synthetic skin constituents and mathematical curve-fitting models. It has been observed that the NMF concentration plays an important role in skin molecular constituents at different depths [11,12,13]. Raman spectroscopy has been extended to understand the changes in skin constituents that are responsible for the pathogenesis of AD. O’Regan et al. discovered that NMF’s Raman signature in the SC can be used as a marker of the FLG genotype in patients with moderate-to-severe AD and that NMF content can even further differentiate among them [6]. Miltz et al. measured lipid and water concentrations other than NMF in the stratum corneum using Raman spectroscopy in a French population and genotyped for the major European FLG mutation. It was found that low concentrations of specific skin constituents such as histidine, alanine, glycine, and pyrrolidone-5-carboxylic acid in the SC was associated with FLG mutations with 92% specificity [7]. Janssens et al. first found that an increase in short chain length ceramides led to the aberrant lipid organization in the SC and hence to an impaired skin barrier function in patients with eczema. Later, they reported that the lipid/protein ratio in patients with eczema is related to skin barrier function and it is proportional to the dry SC mass per skin area in lesioned SC of patients with eczema [8,9]. Verzeaux et al. identified a modification of lipid organization and conformation in addition to the decrease in the lipid-to-protein ratio using ordinary partial least square (OPLS) regression in atopic skin [10]. Recently, Ho et al. used a support vector machine (SVM) model to derive an Eczema Biochemical Index (EBI) to further stratify the severity of AD patients based on the skin constituents’ content measured using a handheld CRM system [14].

The filaggrin mutation or the loss of skin ceramides resulting in skin barrier dysfunction has been studied extensively. However, in the previously reported eczema clinical studies using CRM, machine learning models were not utilized to understand the underlying chemical biomarker information in terms of their characteristic Raman wavenumber or wavebands. In this work, we report the use of machine learning methods for discrimination or classification analysis between patients with eczema and healthy subjects from the Raman signatures acquired within the fingerprint wavenumber region. Machine learning classification results from multiple supervised machine learning algorithms are reported from the Raman eczema spectral dataset to develop an accurate and robust classification model. For optimum performance of the partial least squares discriminant analysis (PLS-DA) classification model, the minimum required number of latent variables (LVs) were evaluated while preserving the least classification error. Further, the classification model was evaluated using the stratified K-fold cross-validation method and computing classification metrics such as confusion matrix, sensitivity, specificity, etc. The detailed Raman spectra analysis in terms of wavenumber contribution responsible for the discrimination between two subject groups is presented with the help of a variable importance in projection (VIP) score developed using the PLS-DA classification model, which can be further utilized for the variable selection to improve classification accuracy.

## 2. Materials and Methods

In this study, patients with atopic dermatitis (*n* = 52) and healthy subjects (*n* = 20) with no other known skin disease participated after initial consultation with clinician. The disease severity of the eczema patients was evaluated by using scoring of atopic dermatitis (SCO-RAD) after their routine checkup by the clinician. A total of 8 mild (SCO-RAD < 25), 31 moderate (25 < SCO-RAD < 50), and 13 severe (SCO-RAD > 50) eczema subjects participated in the clinical study. Since the skin pigmentation reduces the signal-to-noise ratio of the acquired Raman signal, volar arm location with visible lesion is preferred for data acquisition. Additionally, subjects with a Fitzpatrick (FP) score either 3 or 4 were preferred for data acquisition. Among all the 52 eczema subjects, 12 subjects with an FP score of 3, 38 subjects with an FP score of 4, and 2 subjects with an FP score of 5 were recruited. On the other hand, among 20 healthy subjects, 11 subjects with an FP score of 3, 5 subjects with an FP score of 4, and 4 subjects with an FP score of 5 were recruited. Skin physiology measurements such as TEWL were acquired and used for power analysis to substantiate each group sample size. The mean ± standard deviation of TEWL for the eczema and healthy subject group was 17.77 ± 9.0891 and 10.58 ± 2.7964, respectively. The two-sample *t*-test of the respective two group populations’ TEWL value confirmed that the sample size of two groups was sufficient to be differentiated with *p* < 0.001. All participants were above age 21 at the time of recruitment and did not apply any dermatological products to their forearms prior to the clinical study. All the Raman spectroscopy data were processed using open-source Python programming language version 3.8.6 with additional routines and libraries through the Anaconda distribution. The clinical study was approved by the National Healthcare Group Domain Specific Review Board (DSRB reference number 2017/00932) and informed consent was obtained from all the participants. The clinical study involving human participants was performed in accordance as approved by the Institutional Review Board.

### 2.1. In Vivo Non-Invasive Raman Micro-Spectroscopy

**Figure 1:**
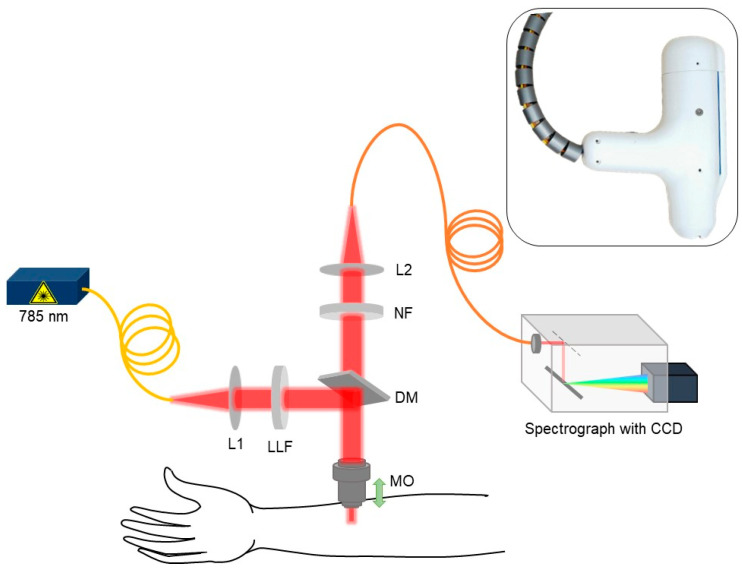
Schematic for skin in vivo CRM system (L1 and L2: beam expander, LLF: laser line filter, MO: microscopic objective, DM: dichroic mirror, and NF: notch filter).

**Figure A1:**
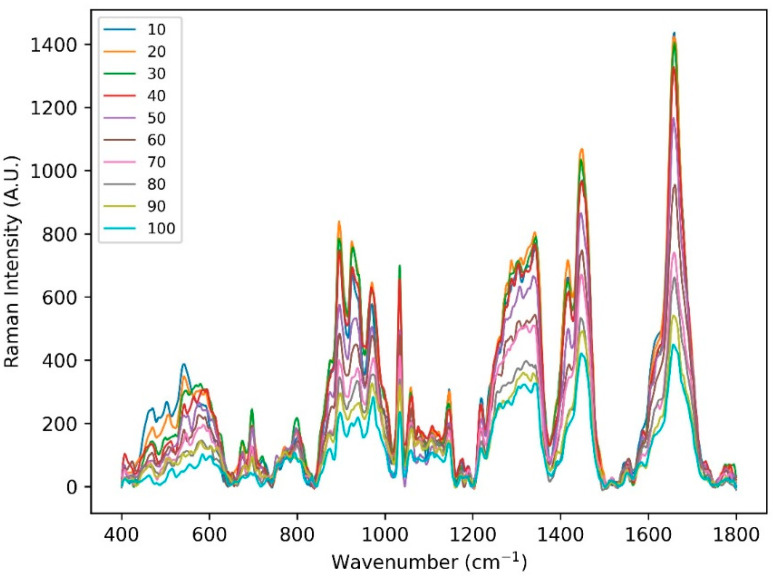
Raman spectral acquired from one of the healthy subject’s volar arm locations using handheld CRM system in the fingerprint region at 10 different depths (10 to 100 µm).

An in-house handheld CRM system was developed to conduct the eczema clinical trial by measuring the Raman spectra at the lower volar arm location in vivo as shown in the schematic in Figure 1. A 3D-printed fixture was fabricated to hold and rest the arm while acquiring the data from the handheld CRM system. The skin surface was illuminated using a 785 nm single-mode fiber-coupled laser (Innovative Photonic Solutions U-type, Innovative Photonic Solutions, Inc., Plainsboro, NJ, USA) and a laser line filter (Chroma 49950-RT, Chroma Technology Corp, Bellows Falls, VT, USA) with a laser power of ≈ 25 mW on the skin surface after passing through a microscopic objective (Nikon CFI Plan Fluor 40×/0.75, Nikon Corporation, Tokyo, Japan). In order to avoid direct contact between the microscopic objective and subject’s skin, a thin glass window (cover glass) was placed in the handheld CRM system housing. The cover glass came in direct contact with the patient’s skin and allowed the z-motion of the microscopic objective. The scattered Raman signal was collected using the same microscopic objective and was focused back to the multimode optical fiber with the help of beam expander and relayed to the spectrograph (Andor Kymera 193i, Andor, Belfast, UK) coupled with a charged coupling device (Andor iDus 416, Andor, Belfast, UK). The spectrograph grating (830 lines/mm blazed at 820 nm) and the back-illuminated deep depleted charge coupling device (CCD) camera were chosen in such a way to have the maximum collective quantum efficiency of ≈ 90% in the wavelength region of 800–900 nm where most of the skin Raman spectra was acquired while achieving spectral resolution of ≈0.3 nm. With the help of the stepper motor embedded inside the CRM system, Raman spectra as a function of depth were acquired in vivo at 10 depths at a step size of 10 µm (Appendix A, Figure A1). The Raman spectra acquired between 400 cm^−1^ to 1800 cm^−1^ with unique 1384 intensity data points per subject were further used for spectral preprocessing, normalization, and chemometric machine learning analysis.

### 2.2. Raman Spectra Preprocessing

**Figure A2:**
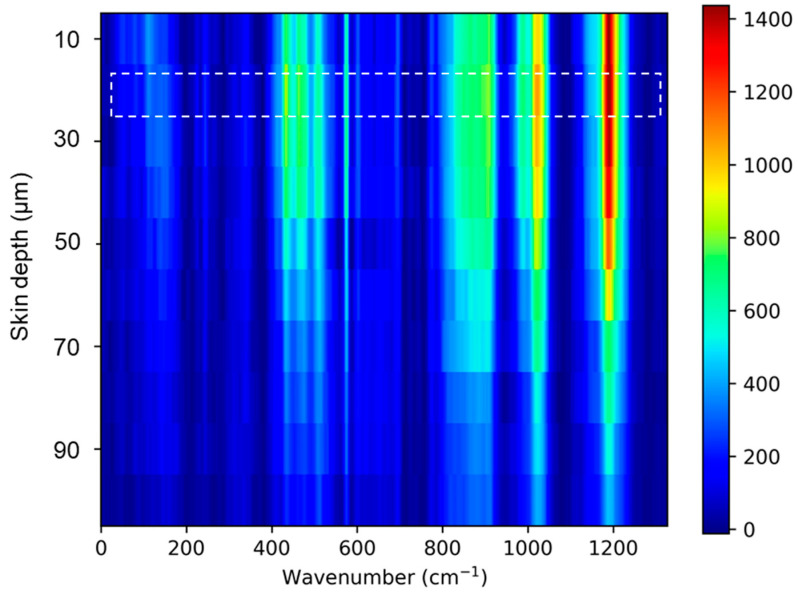
Skin Raman spectra acquired at 10 depths. The skin surface (highlighted) was evaluated by finding Raman spectra maximum peak intensity related to amide I (Keratin) 1655 cm^−1^.

Endogenous tissue autofluorescence and background scattering adds noise to the acquired Raman spectra, masking the important spectral information related to the tissue under investigation. This unwanted noise signal may cause deviation from the linear relationship between the acquired Raman intensity signal and molecular concentration of skin constituents [15,16], warranting the preprocessing of Raman spectra. In the present work, we used the asymmetric least squares (AsLS) baseline correction method to remove the baseline of Raman spectra acquired at all depths [17]. The baseline-corrected Raman spectra at each depth were further processed with the Savitzky–Golay spectral smoothing algorithm to improve the signal-to-noise ratio [18]. After the spectra baseline correction and smoothing, Raman spectra acquired at different depths above and below the skin surface were further analyzed to observe the skin surface precisely. Keratin is an important (≈80% dry weight) component of the skin’s top layer, i.e., SC. It was reported earlier that the keratin contribution of the Raman spectra at 1655 cm^−1^ can help to decipher skin surface information [19]. In our analysis, the skin surface was determined by locating the position of the maximum of keratin amide I intensity profile at 1655 cm^−1^. Thus, the Raman spectra associated with the skin surface were determined from 10 Raman spectra acquired at different depths below the skin surface (Appendix A, Figure A2). The same procedure of preprocessing and skin surface determination was repeated for Raman spectra acquired on all healthy (*n* = 20) and eczema subjects (*n* = 52) and further used as a combined Raman spectral dataset.

### 2.3. Machine Learning Methods

Prior to any chemometric analysis, Raman spectra related to each subject’s volar arm skin surface were standardized using the standard normal variate normalization method. For dimensionality reduction of the Raman spectral dataset, an unsupervised principal component analysis (PCA) was employed to extract a set of orthogonal principal components (PCs) that accounted for the maximum variance in the Raman spectral dataset. However, the first five PCs accounted for a relatively low explained variance (≈65%), which suggested that the features in the Raman spectral dataset for all the subjects could not be explained with few orthogonal dimensions. These PCs did not show a clear demarcation between the two subject groups and thus, the related PC loadings could not deduce the underlying spectral biomarkers in terms of the wavenumber for subject group class differentiation. Since the variance in the Raman spectral dataset could not be explained in fewer principal components, there is a need for other supervised machine learning methods for Raman spectral dataset dimensionality reduction and its binary classification. 

Other supervised machine learning methods such as linear discrimination analysis (LDA), logistic regression, naïve Bayes, K-nearest neighbor (KNN), SVM, and PLS-DA were explored as binary classification models for the preprocessed, normalized Raman spectral dataset. Among all these exploratory supervised machine learning methods, PLS-DA was preferred for further analysis of the Raman eczema spectral dataset because it not only works as a multivariate dimensionality reduction tool like the PCA but also functions as a binary classifier for a dataset with many variables (wavenumbers). The basic principle behind the supervised PLS-DA classifier has been described elsewhere [20,21,22]. In our analysis of the Raman spectral dataset, the correct number of LVs were determined to further enhance the classification accuracy of the PLS-DA classification model to discriminate between the healthy and eczema subject groups. The accuracy of this classification model was verified using the stratified K-fold cross-validation method, as this method allowed us to preserve the percentage of the same sample size from both healthy and eczema groups into two subsets for training and test. Based on this cross-validation, the performance of the classification model was evaluated using binary classification metrics such as classification accuracy, sensitivity, specificity, and the receiver operating characteristics (ROC) curve.

### 2.4. Variable Importance in Projection

The VIP score is an important parameter that can be evaluated from the PLS-DA classification model and estimates the importance of each variable (wavenumber). The VIP score is the weighted sum correlation between PLS latent variables. Variables with a VIP score value greater than the numerical value of one (1) are considered as important and can further help to optimize the PLS-DA classification model [22,23]. The VIP score helps to identify important wavenumbers or Raman band regions that are significantly different in two groups under investigation, i.e., the VIP score can be used to discriminate between the two subject groups by selecting certain wavenumbers closely related to underlying skin constituents. With the help of this quantitative VIP scoring through the classification model, the underlying Raman wavebands associated with different skin constituents could be evaluated.

## 3. Results and Discussion

**Figure 2:**
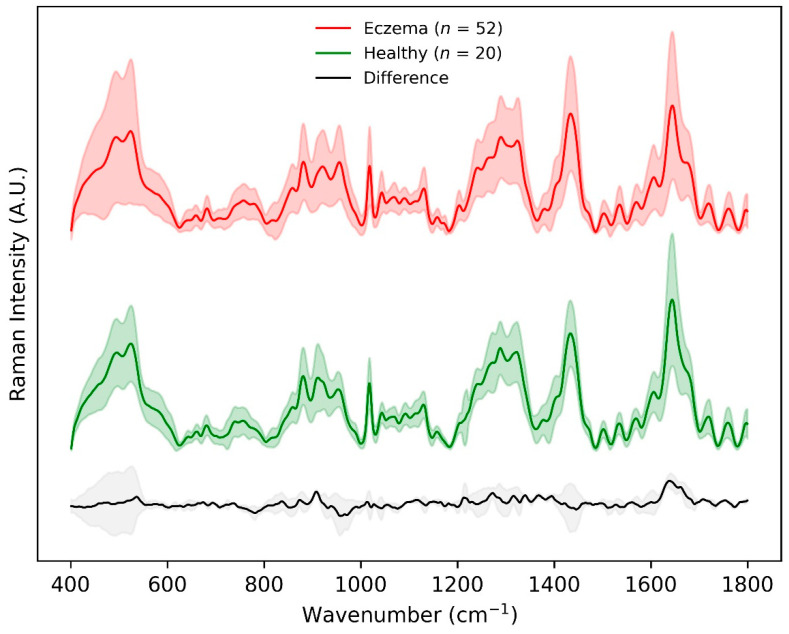
Preprocessed Raman spectra achieved using CRM system within the fingerprint 400–1800 cm^−1^ wavenumber range for eczema (*n* = 52, top) and healthy (*n* = 20, middle) subjects. The difference between the two spectra is shown in grey at the bottom. Shaded region depicts 1 standard deviation variation in the data while the solid line depicts the means of the spectra.

Figure 2 shows the mean ± standard deviation of non-normalized preprocessed skin surface Raman spectra for all healthy (*n* = 20) and eczema subjects (*n* = 52) in the fingerprint region (400–1800 cm^−1^). The major differences in the Raman intensity for the two groups appeared within the wavenumber range 850–930 cm^−1^, amide ⅠⅠⅠ band (1240–1330 cm^−1^), intensity variation of the shoulder at 1420 cm^−1^, and amide Ⅰ band (1640–1680 cm^−1^). This difference was related to the Raman spectra of the uppermost SC layer that was dominated by the vibrational bands of its structural proteins, amino acids, and lipids. The Raman intensity difference within the wavenumber range 850–930 cm^−1^ and shoulder intensity at 1420 cm^−1^ could be attributed to the molecular composition of the SC in terms of NMF that primarily constitutes of amino acids (serine, glycine, alanine, etc.), its derivatives, and pyrrolidone carboxylic acid having a distinctive peak at 885 cm^−1^. The Raman intensity difference in the amide ⅠⅠⅠ band (1240–1330 cm^−1^) was predominantly due to ceramide ⅠⅠⅠ (having one of the distinctive peaks at 1296 cm^−1^), the most abundant lipid in the stratum corneum. The largest mean Raman intensity difference between the two groups was visualized in the amide I band having an intensity peak at 1650 cm^−1^, which corresponds to urocanic acid in the stratum corneum [11,12,13].

These differences in the Raman spectral signature acquired for two groups depict the regions of interest as chemical biomarkers for AD. To further understand the underlying quantitative Raman biomarkers, different supervised binary classification methods were tested for the classification of Raman spectra from the two subject groups. The preprocessed, baseline-corrected, and mean-centered Raman spectral dataset for two subject groups with binary group affinities was used with multiple binary classification methods. Table 1 shows a summary of the results from some of the popular binary classification methods such as LDA, naïve Bayes, logistic regression, KNN, and SVM. The results were evaluated in terms of classification metrics such as aggregated classification accuracy, specificity, sensitivity, and mean ROC AUC score through the stratified K-fold cross-validation method. The stratified cross-validation method maintains the class imbalance in different folds of the training and test Raman spectral dataset. It is evident from these results that these binary classification methods demonstrated good classification accuracy but lacked visualization of classification results in terms of LV score or a scatterplot.

**Figure 3:**
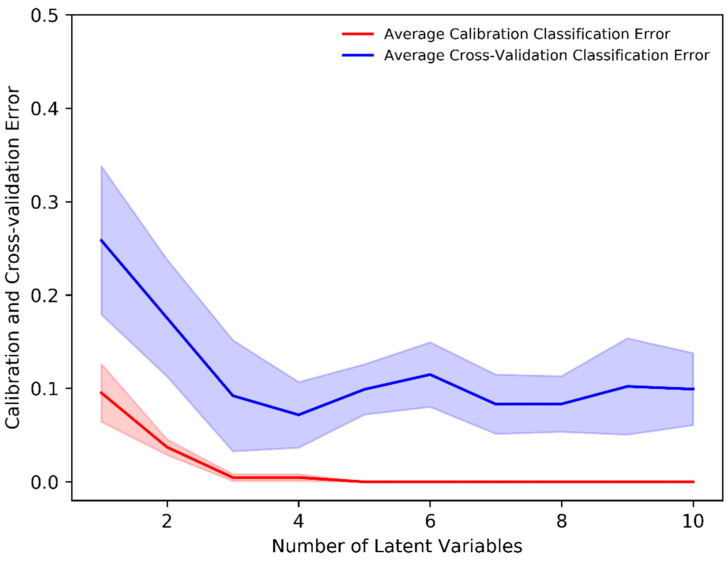
Average calibration and cross-validation classification error evaluated through the stratified K-fold (K = 10) cross-validation in the PLS-DA classification model.

The PLS-DA classification method allows one to visualize the classification between two (or more) subject groups in terms of its LVs and allows for the identification of important variables responsible for classification. The mean-centered, baseline-corrected Raman spectral dataset from the two study groups was used as the descriptor (X) matrix, whereas the response (Y) vector was artificially generated to designate group affinities. The PLS-DA determines the fit between the descriptor matrix and class groups by maximizing the covariance. As a result, LVs in terms of the PLS score were determined. In our analysis, the minimum number of LVs was determined based on minimizing the classification error or improving classification accuracy. Figure 3 shows that a minimum of four (4) LVs was required to build an adequate PLS-DA classification model as the average calibration and cross-validation classification error was ≈0% and 8%, respectively, with four LVs. Thus, a minimum number of four (4) LVs was an ideal number to build a classification model that prevents underfitting or overfitting and imparts highly accurate predictions.

**Figure 4:**
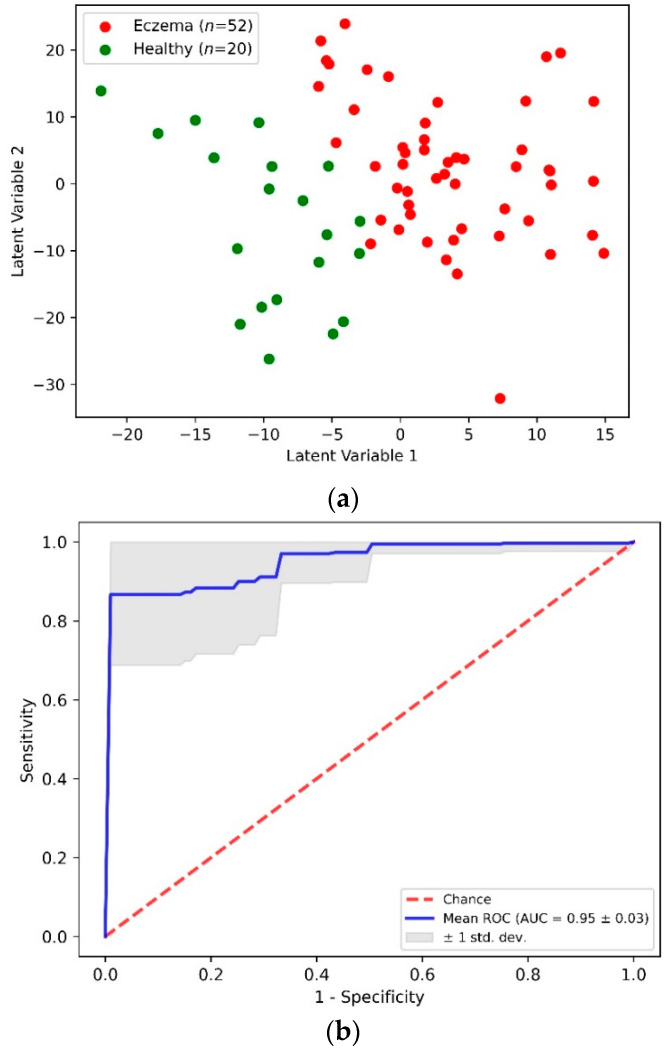
(**a**) Scatter plot of the first two PLS latent variables and (**b**) receiver operating characteristics (ROC) curve from PLS-DA classification of eczema (*n* = 52) and healthy (*n* = 20) subjects.

Figure 4a shows a two-dimensional scatterplot for the first two PLS LVs. This scatterplot shows the clear demarcation between the first two LV values for the eczema and control subject groups. The accuracy of this PLS-DA classification model was determined through the stratified K-fold (K = 10) cross-validation method to nullify any ambiguity due to class imbalance. Table 2 shows an aggregated confusion matrix and related classification metrics evaluated through cross-validation. From the PLS-DA classification model with an accuracy of 0.92 ± 0.05, a sensitivity and specificity of 0.94 and 0.85 was achieved, respectively. Figure 4b shows an averaged receiver operating characteristics (ROC) curve from multiple cross-validation folds that demonstrates the capability of the PLS-DA binary classifier to distinguish between eczema and healthy subjects with threshold variation.

**Figure 5:**
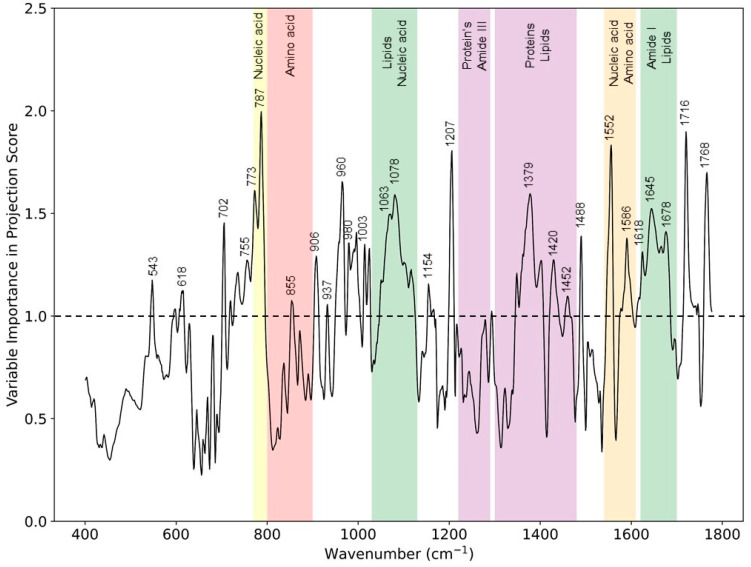
VIP scores from the PLS-DA classification model for eczema (*n* = 52) and healthy subjects (*n* = 20) depicting Raman peaks and wavebands having a numerical value greater than 1.

As shown in Figure 2, the Raman difference spectrum demonstrated a direct comparison between the eczema and healthy subject molecular vibration spectra. As described earlier, this difference spectrum shows that there were certain wavenumber regions where there is a difference in the mean Raman spectral intensity acquired between eczema and healthy subjects. Figure 5 shows the VIP score evaluated using the PLS-DA classification model. A VIP score greater than numerical value one (1) indicates the spectral bands or wavenumbers that are important for optimal PLS-DA classification model performance, i.e., these Raman bands or wavenumbers are discriminatory between the healthy and eczema subject groups. From this figure, it is evident that the most discriminatory wavenumber bands appeared in the lipid along with protein and nucleic acid band (1030 to 1130 cm^−1^, 1300 to 1450 cm^−1^, and 1620 to 1700 cm^−1^) regions of the skin biomolecular composition [24]. Thus, the Raman wavenumber bands attributed to lipids (960, 980, 1078, 1379, and 1655), proteins (618, 755, 855, 980, 1003, 1154, 1207, 1552, and 1655), amino acids (855, 1420, 1452, 1586, and 1716), and nucleic acids (787, 1078, and 1452) could be observed as the most important spectroscopic signatures for the classification between the eczema and healthy subject group. These Raman wavenumbers and wavebands are also tabulated in Table 3 with the peak assignments.

Skin barrier function lipids in terms of SC ceramides have certain spectral features, and changes in these spectral regions have been studied with respect to hydration. These spectral features related to the skin barrier lipid function are ν (C-C) skeletal optical mode (1050–1140 cm^−1^), CH_2_ scissoring mode (1410–1480 cm^−1^), and ν (C=O) amide I band (1600–1650 cm^−1^) in the fingerprint region [6,29,30]. These spectral bands are related to organization and conformation, as well as the polar interactions of the SC lipids, and any change in these bands can help to elucidate the effect the atopic dermatitis on the skin barrier function. These spectral regions related to skin barrier function lipids correlate to our finding from this study in terms of the VIP score of important spectral regions (1030 to 1130 cm^−1^, 1350 to 1450 cm^−1^, and 1620 to 1680 cm^−1^). Thus, the VIP score from the PLS-DA classification model allows one to evaluate important chemical biomarkers that are differentiators between two study groups indicating a reduction in ceramides in an impaired SC that affects the barrier function of the skin, a hallmark of AD. 

## 4. Conclusions

Non-invasive quantitative analysis of AD in terms of skin molecular composition is important for dermatological diagnosis and treatment. Quantitative cognition of skin bio-molecular composition can be accomplished using CRM; however, multivariate analysis is needed to elucidate complex Raman spectroscopic signatures. Here, we presented a method to classify between AD and healthy subjects based on multivariate analysis using PLS-DA and CRM. Thus, the PLS-DA classification model is currently limited for binary classification; however, it would be interesting to evaluate multiclass classification based on eczema disease severity. Our approach to use the PLS-DA classification method permitted dimensional reduction, classification, and variable selection for Raman micro-spectroscopy data. We cross-validated our PLS-DA classification model and achieved a sensitivity and specificity of 0.94 and 0.85, respectively. The classification accuracy of the PLS-DA model can be further enhanced by selecting only the wavenumber bands having a VIP score ≥1. Further, with the help of the VIP score, important Raman spectroscopic signatures in terms of Raman peaks or wavenumber bands for lipids, proteins, and nucleic acids were evaluated that can act as biomarkers to assess the skin condition for eczema subjects in clinics and further therapeutics. This quantitative analysis of skin inflammatory conditions such as AD using CRM and multivariate analysis may pave the way for next-generation diagnosis unlike the current subjective scoring assessments used in clinics. 

## Figures and Tables

**Table 1:** Quantitative comparison of different binary classification methods in terms of classification evaluation metrics. Stratified K-fold (K = 10) cross-validation method was used to evaluate the aggregated metrics.

Classification Method	Number of PC Used	Aggregated Classification Accuracy	Sensitivity	Specificity	Mean ROC AUC Score ^1^
PCA + LDA	18	0.84 ± 0.05	0.87	0.80	0.83 ± 0.14
PCA + Logistic Regression	14	0.82 ± 0.06	0.87	0.70	0.78 ± 0.14
PCA + Naïve Bayes	6	0.74 ± 0.07	0.83	0.50	0.66 ± 0.16
PCA + Naïve Bayes (nearest neighbors = 4)	17	0.79 ± 0.07	0.88	0.55	0.72 ± 0.17
PCA + Support Vector Machine	11	0.84 ± 0.06	0.87	0.75	0.81 ± 0.16

^1^ ROC AUC Score—Area Under Receiver Operating Characteristics Curve.

**Table 2:** Classification metrics from the PLS-DA binary classification model using the stratified K-fold (K = 10) cross validation method for eczema (*n* = 52) and healthy (*n* = 20) subjects.

PLS-DA Classification Metrics (Stratified K-Fold (K = 10) Cross Validation)		
Confusion Matrix	17	3
	3	49
Accuracy	0.92 ± 0.05	
Sensitivity	0.94	
Specificity	0.85	
AUC	0.95 ± 0.03	

**Table 3:** Assignment of most prominent Raman wavenumbers and bands deduced from VIP score depicting difference between healthy and eczema skin. *ν* = stretch, *δ* = deformation.

Peak Position (in cm^−1^)	Vibrational Mode Assignment	Associated References
543	*ν* (SS), Cholesterol	[25,26,27]
618	*ν* (C-C) twisting (protein)	[25,26]
702	Cholesterol	[24,25,26,27,28]
755	Symmetric breathing of tryptophan	[25,26]
773	*ν* (C-C) ring breathing	[25]
787	Nucleic acid	[25]
855	*ν* (C-C) of proline, *δ* (CCH) of protein	[24,25,26,27,28]
906	Tyrosine (Amino acid)	[25]
937	ν (C-C) *α*-helix conformation (protein)	[24,25,26,27,28]
960	*δ* (CCH) olefinic	[24]
980	ν (C-C) stretching *β*-sheet (proteins), CH bending (lipids)	[25,26]
1003	*ν* (C-C) ring breathing of phenylalanine	[24,25,26,27,28]
1063	*ν* (C-C) skeletal stretching (lipid)	[24,25,26,27,28]
1078	*ν* (C-C) or *ν* (C-O), phospholipids (lipid)	[25,26,28]
1154	*ν* (C-C), *δ* (COH) (lipid)	[24,26,27]
1207	Tryptophan, phenylalanine (protein)	[24,25,27]
1379	*δ* (CH_3_) Symmetric (lipid)	[24,25]
1420	*ν* (C=O) of COO^−^ (amino acid aspartic and glutamic acid)	[24,26]
1452	*δ* (CH_3_), *δ* (CH_2_) (Proteins and lipids)	[24,25,26,27,28]
1552	*δ* (NH) and *ν* (CN) amide II (protein)	[24,25]
1586	*ν* (C=C) olefinic (protein)	[24,25,26]
1618	*ν* (C=C) Tryptophan (protein)	[25,27]
1645	(O-H) Water and amide I (*α*-helix) (protein)	[24,25]
1655–1680	*ν* (C=O) amide I, amide I (*α*-helix), lipid *ν*(C=C) (proteins and lipids)	[24,25,26,27,28]
1716	*ν* (C=O) OH (amino acid aspartic and glutamic acid)	[25,26]
1768	*ν* (COO)	[24]

## Data Availability

Not applicable.
